# Methacrylated Gelatin as a Scaffold for Mechanically Isolated Stromal Vascular Fraction for Cutaneous Wound Repair

**DOI:** 10.3390/ijms241813944

**Published:** 2023-09-11

**Authors:** Mauro Vasella, Kevin Arnke, Dalia Dranseikiene, Elia Guzzi, Francesca Melega, Gregory Reid, Holger Jan Klein, Riccardo Schweizer, Mark W. Tibbitt, Bong-Sung Kim

**Affiliations:** 1 Department of Plastic Surgery and Hand Surgery, University Hospital Zurich, 8091 Zurich, Switzerland; mauro.vasella@usz.ch (M.V.); gregory.reid@usz.ch (G.R.); 2 Center for Preclinical Development, University Hospital Zurich, 8091 Zurich, Switzerland; kevin.arnke@usz.ch; 3 Macromolecular Engineering Laboratory, Department of Mechanical and Process Engineering, ETH Zurich, 8092 Zurich, Switzerland; ddranseik@ethz.ch (D.D.); guzzie@ethz.ch (E.G.); mtibbitt@ethz.ch (M.W.T.); 4 Institute of Pathology and Molecular Pathology, University Hospital Zurich, 8091 Zurich, Switzerland; francesca.melega@usz.ch; 5 Department of Plastic Surgery and Hand Surgery, Cantonal Hospital Aarau, 5001 Aarau, Switzerland; holger.klein@ksa.ch; 6 Department of Plastic, Reconstructive and Aesthetic Surgery, Regional Hospital Lugano, 6900 Lugano, Switzerland; riccardo.schweizer@gmail.com

**Keywords:** hydrogel, GelMA, natural compound, adipose-derived stromal cells, SVF, regenerative medicine

## Abstract

Mechanically processed stromal vascular fraction (mSVF) is a highly interesting cell source for regenerative purposes, including wound healing, and a practical alternative to enzymatically isolated SVF. In the clinical context, SVF benefits from scaffolds that facilitate viability and other cellular properties. In the present work, the feasibility of methacrylated gelatin (GelMA), a stiffness-tunable, light-inducible hydrogel with high biocompatibility is investigated as a scaffold for SVF in an in vitro setting. Lipoaspirates from elective surgical procedures were collected and processed to mSVF and mixed with GelMA precursor solutions. Non-encapsulated mSVF served as a control. Viability was measured over 21 days. Secreted basic fibroblast growth factor (bFGF) levels were measured on days 1, 7 and 21 by ELISA. IHC was performed to detect VEGF-A, perilipin-2, and CD73 expression on days 7 and 21. The impact of GelMA-mSVF on human dermal fibroblasts was measured in a co-culture assay by the same viability assay. The viability of cultured GelMA-mSVF was significantly higher after 21 days (*p* < 0.01) when compared to mSVF alone. Also, GelMA-mSVF secreted stable levels of bFGF over 21 days. While VEGF-A was primarily expressed on day 21, perilipin-2 and CD73-positive cells were observed on days 7 and 21. Finally, GelMA-mSVF significantly improved fibroblast viability as compared with GelMA alone (*p* < 0.01). GelMA may be a promising scaffold for mSVF as it maintains cell viability and proliferation with the release of growth factors while facilitating adipogenic differentiation, stromal cell marker expression and fibroblast proliferation.

## 1. Introduction

Impaired wound healing and chronic wounds are a major healthcare burden both financially and clinically, leading to increased costs, considerable morbidity and mortality, prolonged hospital stays, and reduced quality of life.

The wound healing process is coordinated by a series of factors and is defined in different phases including hemostasis, inflammation, proliferation, maturation, and remodelling [1,2]. Delayed wound healing and chronic wounds are often associated with underlying conditions due to local and/or systemic causes, such as ischemia, diabetes, vascular disease, and obesity, among others, possibly resulting in serious local or systemic complications and even death [3].

Advances in wound care through regenerative medical approaches, such as the delivery of growth factors or stromal cells guided by tissue-engineering concepts, offer promising solutions for improving outcomes [4]. Regenerative medicine is particularly relevant in scenarios where local or systemic factors prohibit invasive surgery. Autologous fat grafting, adipose-derived stromal cells (ASCs), and stromal vascular fraction (SVF) have recently emerged as potential therapeutic approaches [5]. A large body of literature provides evidence for the clinical efficacy of ASC/SVF in wound healing, including preclinical [6], clinical [7] and randomized control trials [8].

Standard processing of ASCs via collagenase digestion and subsequent cell culture is time-consuming and does not permit immediate re-injection in a single-stage procedure. To overcome these limitations, clinicians have proposed various protocols for mechanical SVF (mSVF) isolation. In this context, we introduced a protocol consisting of emulsification and two-step centrifugation resulting in a so-called lipoconcentrate [9]. While direct application of mSVF to wounds is a possibility for improved healing, the residence time on the wound is limited. Research in the field of tissue engineering has demonstrated the benefits of a scaffold for cell therapeutics that can mimic the natural extracellular matrix, provide mechanical support, and promote nutrient and oxygen transport to improve tissue integration, cell migration, proliferation, and differentiation [10].

Hydrogels are particularly attractive scaffolds for wound healing applications. They are three-dimensional, cross-linked networks of hydrophilic polymers capable of absorbing and retaining large amounts of water and/or other biological fluids [10]. They can be categorized by features such as their origin (natural, synthetic or semi-synthetic) and gelation method, which can be physically or chemically induced [11]. Hydrogels are commonly used in biomedical applications because of their ability to recapitulate critical features of the extracellular matrix of tissues and their biocompatibility [12]. Moreover, hydrogels can promote the proliferation of cells [13] and be tuned to exhibit mechanical properties similar to tissues in the human body [14]. Huang et al. have thoroughly reviewed the current literature regarding hydrogels for the differentiation of ASCs, underlining the enormous potential of combining ASCs with hydrogels in the regeneration of different tissues [10].

Methacrylated gelatin (GelMA) is a semi-synthetic hydrogel composed of modified gelatin with methacrylate groups, making it cross-linkable by photopolymerization. Importantly, GelMA is biocompatible as well as biodegradable [15]. It provides a three-dimensional microenvironment that mimics the extracellular matrix and supports cell growth, adhesion, migration and differentiation [16]. Photopolymerization of GelMA using a portable light source is an interesting feature as it allows quick and practical customization of the mSVF–hydrogel mix to the three-dimensional needs of the wound [17].

Herein, we present the first investigation of the feasibility and potential of GelMA as a scaffold for mechanically processed SVF, a novel cell tissue source that is more practical and circumvents existing regulatory concerns in an in vitro setting to examine its impact on cell viability, proliferation, and its future application in advanced cell-based wound therapies. 

## 2. Results

### 2.1. Patients

Samples were collected from 10 patients (9 female, 1 male). Mean age was 43.2 years (range 18–65). Mean weight was 75.63 kg (range 63–87.5). Mean BMI was 27.9 kg/m^2^ (range 21.6–34.6). Patient demographics are summarized in Table 1.

### 2.2. Viability Assay

**Figure 1:**
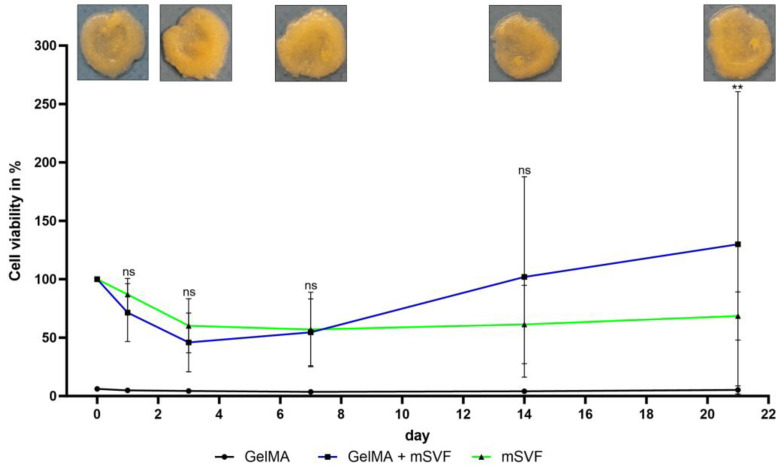
AB viability assay over a period of 21 days with documentation of the GelMA-mSVF constructs at 0, 1, 3, 7, 14 and 21 days, demonstrating an initial drop-off of viability after mSVF processing and photocross-linking. After 21 days the viability of the GelMA-mSVF compared to mSVF alone was significantly higher. “ns” signifies non-significant. ** signifies *p* ≤ 0.01.

Viability at day 0 was defined as reference (100%) and initially dropped to a mean of 46% for the GelMA–mSVF and 60% for the PC group at day 3. Afterwards, there was a steady increase of viability with the highest mean value of 130% being reached in the GelMA–mSVF group and significant when compared to PC on day 21 (mean (PC; d21) 69 ± 7; (GelMA–mSVF; d21) 130 ± 46; *p* = 0.0093). The difference was non-significant for day 1 (mean (PC; d1) 87 ± 5; (GelMA–mSVF; d1) 72 ± 9; *p* = 0.7331), day 3 (mean (PC; d3) 60 ± 8; (GelMA–mSVF; d3) 45 ± 9; *p* = 0.7652), day 7 (mean (PC; d7) 57 ± 11; (GelMA–mSVF; d7) 55 ± 10; *p* = 0.9919), and day 14 (mean (PC; d14) 61 ± 12; (GelMA–mSVF; d14) 102 ± 30; *p* = 0.121). Results are illustrated in Figure 1.

### 2.3. ELISA

**Figure 2:**
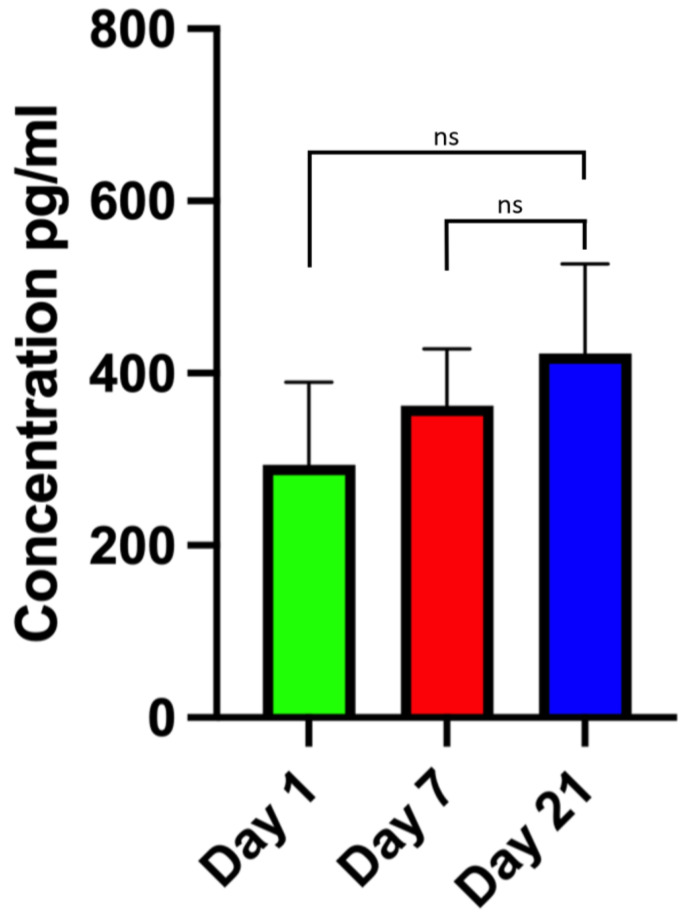
ELISA of secreted bFGF levels in GelMA-mSVF samples on days 1, 7 and 21 showing a steady increase of secretion, however, not in a significant matter. “ns” signifies non-significant.

Levels of secreted bFGF as a marker for cell growth and angiogenesis in wound repair were measured in the GelMA–mSVF group on days 1, 7, and 21 in pg/mL. There was a steady increase of bFGF secretion; however, none of the values were significant when all days were compared to each other (mean (d1) 293.4 ± 96.3 and (d7) 362.1 ± 66.0; *p* = 0.9983); (mean (d1) 293.4 ± 96.3 and (d21) 422.8 ± 104.1; *p* = 0.9723); (mean (d7) 362.1 ± 66.0 and (d21) 422.8 ± 104.1; *p* = 0.9994). Results are illustrated in Figure 2.

### 2.4. Histology, IHC and IF

**Figure 3:**
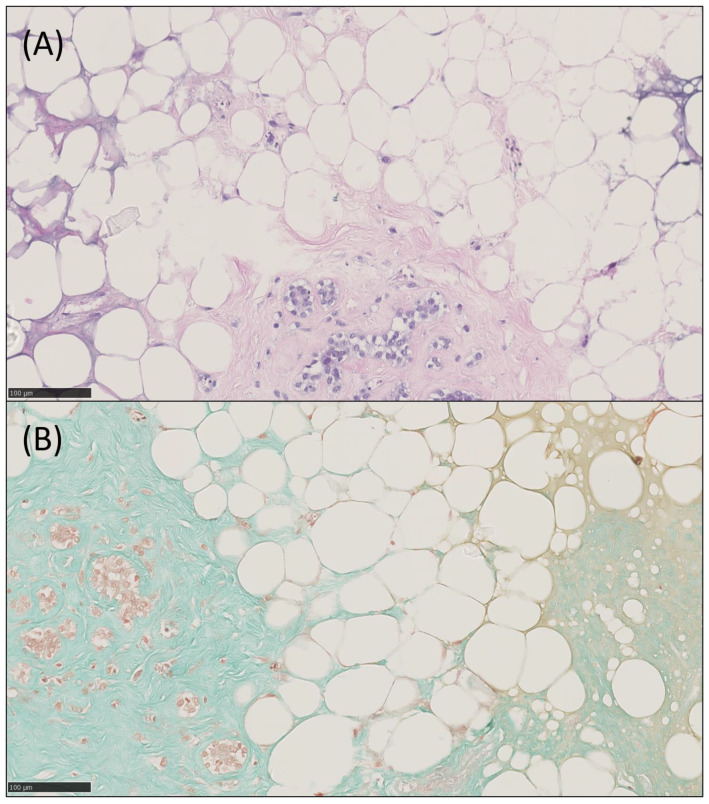
HE (**A**) and trichrome staining (**B**) of a GelMA-mSVF sample at day 21. Overall demonstration of cell cluster formation, adipose and connective tissue which is supported by the surrounding GelMA. Collagen fibres are stained green in (**B**). Scale bars, 100 µm.

**Figure 4:**
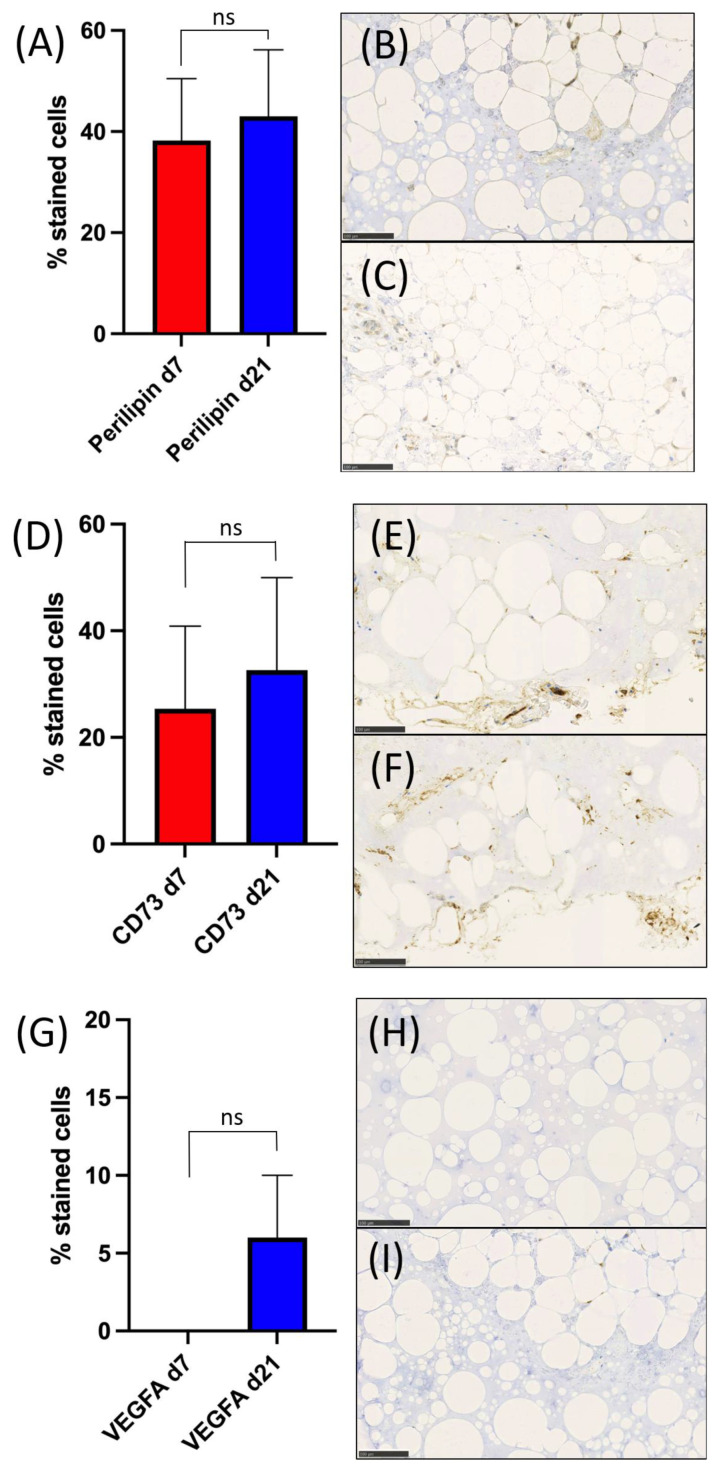
Comparison of the percentage of positive cells in GelMA-mSVF in culture and growth medium on days 7 and 21 by IHC staining of Perilipin-2 (**A**), VEGF-A (**D**), CD73 (**G**). (**B**,**C**) Perilipin-2 staining showing progressive cell cluster formation, adipose tissue and slightly increased expression when day 7 (**B**) was compared to day 21 (**C**). (**E**,**F**) CD73 staining illustrating elevated staining intensity and cell clusters for both days 7 (**E**) and 21 (**F**) with a slight increase in positive cells for the latter. (**H**,**I**) VEGF-A staining visualizing a marginal increase of positive cells in between adipocytes for day 21 (**I**) when compared to day 7 (**H**). Scale bars, 100 µm. “ns” signifies non-significant.

Histopathological analysis of HE, trichrome, VEGF-A (vascularization and angiogenesis), CD73 (mesenchymal stem cells and endothelial cells), and Perilipin-2 (adipogenesis) of days 7 and 21 was performed. HE showed increased numbers of cells, cell clusters and larger areas of stroma and adipose tissue including adipocytes. Trichrome staining visualized the increase of connective tissue fibres, which were predominantly green, indicating the presence of collagen fibres. IHC of HE and trichrome are demonstrated in Figure 3. Nevertheless, there was no significant difference in staining intensity for the antibodies against VEGF-A (mean (d7) 0 ± 0; (d21) 0.6 ± 0.9; *p* = 0.208), CD73 (mean (d7) 1.3 ± 0.3; (d21) 1.6 ± 0.4; *p* = 0.208), and Perilipin-2 (mean (d7) 1.2 ± 0.5; (d21) 1.4 ± 0.5; *p* = 0.3739). The number of positive cells for VEGF-A, CD73, and Perilipin-2 was higher on day 21 when compared to day 7; however, again, the values were non-significant for VEGF-A (mean (d7) 0 ± 0; (d21) 0.05 ± 0.09; *p* = 0.605), CD73 (mean (d7) 0.25 ± 0.35; (d21) 0.33 ± 0.39; *p* = 0.06245), and Perilipin-2 (mean (d7) 0.38 ± 0.27; (d21) 0.43 ± 0.29; *p* = 0.6322). Scores of all patients and representative IHC images are summarized in Figure 4.

**Figure 5:**
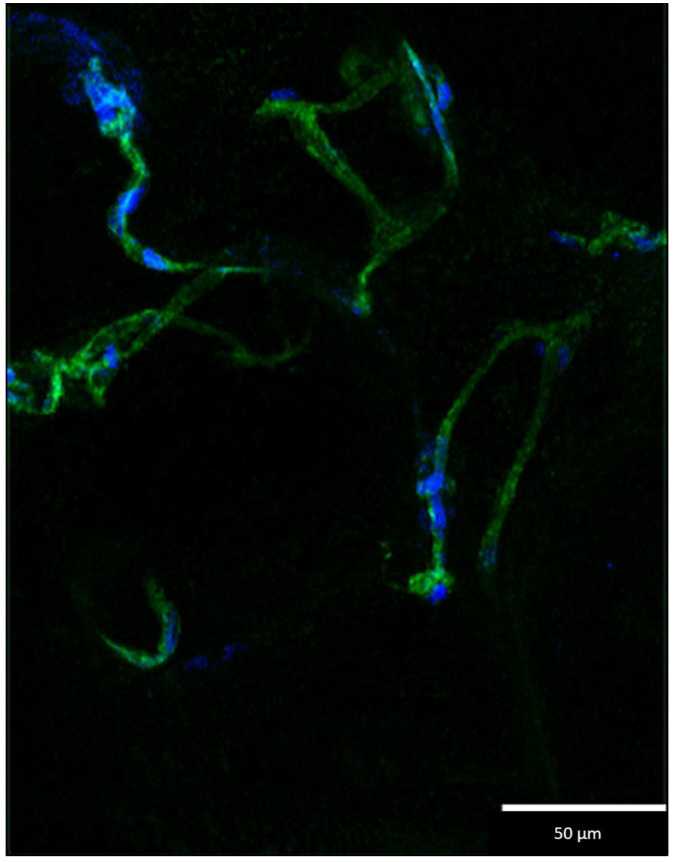
DAPI/phalloidin staining of GelMA-mSVF at day 3 visualizing viable cell nuclei (blue) and actin filaments (green). The cells have a spread morphology and grow along the cytoskeleton.

GelMA-mSVF were positively stained for DAPI and phalloidin. Beyond enhanced staining of viable cell nuclei and actin filaments, cells tended to assume spread morphology as well as growth along the cytoskeleton over time. DAPI and phalloidin staining on day 3 is illustrated in Figure 5.

### 2.5. Co-Culture Assay

**Figure 6:**
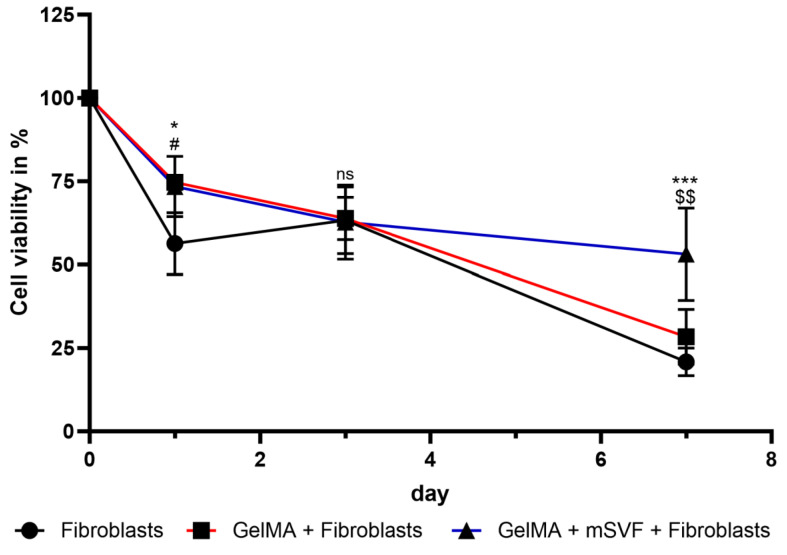
Viability of GelMA-mSVF in a co-culture assay with fibroblasts in starvation medium. Viability was significantly higher in the GSF and GF group on day 1 when compared to FB control group. More importantly, viability was significantly higher on day 7 for the GSF when compared to either GF or FB, indicating that GelMA helped the cells survive longer in a challenging environment. “ns” indicates non-significant. * indicates *p* ≤ 0.05 and *** *p* ≤ 0.0001 for fibroblasts vs. GelMA-mSVF fibroblasts. # indicates *p* ≤ 0.05 for fibroblasts vs. GelMA fibroblasts. $$ indicates *p* ≤ 0.01 for GelMA fibroblasts vs. GelMA-mSVF fibroblasts.

GelMA–mSVF mixes were co-cultured with dermal fibroblasts to study the potential dermal wound repair capacity of GelMA–mSVF. Fibroblast viability at day 0 was defined as 100% and served as a reference. Viability showed a steady decrease over the course of 7 days. Nevertheless, viability was significantly higher for fibroblasts with both GelMA–mSVF and GelMA when compared with the control group at day 1 (mean (fibroblast; d1) 56 ± 5; (GelMA-mSVF; d1) 73 ± 5; *p* = 0.0309; mean (fibroblast; d1) 56 ± 5; (GelMA; d1) 75 ± 1; *p* = 0.0184) and day 7 when comparing GelMA–mSVF with the control group and GelMA alone (mean (fibroblast; d7) 21 ± 2; (GelMA-mSVF; d7) 53 ± 8; *p* ≤ 0.0001; mean (GelMA; d7) 28 ± 5; (GelMA-mSVF; d7) 53 ± 8; *p* = 0.0011). Results are shown in Figure 6.

## 3. Discussion

Given regulatory boundaries, costs and the time-consuming process of enzymatic SVF isolation, mSVF protocols have emerged as a practical alternative for plastic surgeons. The strong wound healing properties of mSVF, despite the decreased cell load [18], are probably due to the fact that the mechanical processing technique is capable of preserving the natural extracellular matrix of the adipose tissue, growth factors, and cellular properties [19].

One fundamental challenge in the topical use of mSVF is its relatively liquescent texture, which is difficult to control in the clinical setting. While the injection or application of mSVF is easy, maintaining mSVF on surfaces, including cutaneous wounds, without risk of dislocation or desiccation requires additional supplementary strategies. Also, mSVF cells may benefit from an additional scaffold to maintain biological properties over time.

GelMA has been proposed as a potential scaffold for SVF due to its biocompatibility, biodegradability, and tunable mechanical properties [10]. Its tunability of physical properties is influenced by its concentration, the concentration of the photoinitiator, and exposure time under UV light, resulting in different levels or degrees of cross-linking [20]. Cross-linking can be achieved even at low temperatures [20], which is handy for its on-site clinical application. The resulting 3D structure mimics aspects of the microenvironment of native tissue and increases cell-to-cell interactions by providing a larger surface area for cell adhesion, enabling the formation of cell clusters [21]. Also, the hydrated nature of the biomaterial can enhance nutrient and oxygen transport, which fosters cell proliferation and migration [22]. There are also opportunities for advanced processing of GelMA, such as 3D bioprinting [23] and implementation of drugs with controlled local release patterns [24]. GelMA can be easily functionalized, e.g., by growth factors or extracellular matrix components, to enhance cell proliferation as well as differentiation, making it a promising scaffold for SVF-based therapies [25]. While previous articles have already shown GelMA in conjunction with enzymatically isolated ASCs, we herein present the first investigation of GelMA as a scaffold for mechanically processed SVF, a cell–extracellular matrix (ECM) mixture with the goal of examining its feasibility in the future application in advanced cell-based wound therapies.

Our viability experiments demonstrated that GelMA–mSVF mixtures improve viability significantly when compared to mSVF alone over an extended period of time. These results are in line with a study by Kessler et al., demonstrating that the viability of ASCs was increased in combination with a GelMA/hyaluronan-based hydrogel as well as increased adipogenesis and angiogenesis in vitro [26]. However, compared to our study, they employed collagenase for the processing of SVF, different stock solutions of GelMA (10 and 20 wt%), different wavelengths for crosslinking (365 nm versus 405 nm), and assessed further characteristics such as swelling and cytotoxicity properties of their hydrogel. Also, the authors used a composite hydrogel composed of GelMA and hyaluronan. We, by contrast, intentionally skipped additional components, as the mSVF itself is not a pure cell mixture but also includes significant amounts of extracellular matrix (ECM) molecules as demonstrated earlier [27]. O’Donnell et al. investigated GelMA as a promising scaffold in an in vitro study reporting increased cell differentiation of ASCs into mature adipocytes when combined with the hydrogel [28]. While the scaffold itself was designed for another research rationale, i.e., osteoarthritis and the design of a 3D structure by a custom bioreactor, our experiments neglected 3D characteristics as they are of secondary interest for wound healing purposes where the key goal is to generate a vascularized tissue layer. However, in accordance with O’Donnell’s observations, our previous studies showed that mSVF has proadipogenic and importantly pro-angiogenic properties [27] that support mSVF as a suitable cell source. O’Donnell and colleagues used the same stock solution of GelMA (15 wt%), while photoactivation was only two minutes compared to five minutes in our approach, most likely due to smaller construct sizes and the more liquid texture of mSVF. More recently, Li et al. demonstrated enhanced wound healing in a mouse model by combining hypoxic pretreated ASCs and GelMA topically, likely to augment levels of VEGF and in turn increase angiogenesis [29]. In Li and colleagues’ study, ASCs retrieved from enzymatic digestion were preconditioned by hypoxia for cell activation and enhancing cytokine release. While we agree that hypoxia exerts favourable effects on ASCs [30], our experimental setup was directed towards the basic combination of GelMA and non-manipulated mSVF, as hypoxic preconditioning would add to hurdles in the clinical translation process. Also, while collagenase-digested and -cultured ASCs have been an integral cell type for some years, the investigation of mechanical protocols for adipose-derived progenitor cell isolation still is in its infancy. Consequently, a stepwise process that may lead to advanced study protocols including additional scaffold components, cell preconditioning or supplementation of bioactive molecules may follow. Colle et al. [31] proposed GelMA enhanced by ASC microchips by seeding on microchips with the goal of building tissue blocks for breast reconstruction. In breast reconstruction, large three-dimensional volumes are challenges that are yet to be overcome as the impressive work of Wayne Morrison delineates [32]. Bioprinting of mSVF, however, may be a cumbersome endeavour due to its consistency and clinically less relevant for cutaneous wounds that are characterized by their surface rather than volume. Several other studies, such as those by Li et al. [33] and Huber et al. [34], proposed other cell sources, i.e., umbilical-cord-derived mesenchymal stromal cells or adipocytes, mixed with GelMA for wound healing and adipose tissue engineering with slightly varying parameters for cell number/GelMA preparation. That being said, all aforementioned published articles still highlight isolated singular cell types, whereas our results indicate that GelMA also is capable of incorporating a more complex cell–tissue blend such as mSVF.

Our IHC staining demonstrated stable expressions of Perilipin-2, CD73, and VEGF-A, as well as increased formation of adipose and connective tissue, suggesting that GelMA supports growth of cells within the heterogenous mSVF mix, adipogenesis, and expression of stromal cell markers. These results are congruent with a study by Wittmann et al., revealing the successful formation of adipose tissue when SVF was mixed with hydrogels [35]. Nevertheless, the group utilized stable fibrin gels, SVF was prepared using a collagenase, and they eventually implanted the SVF-seeded gels in mice for an in vivo approach, when comparing it to our methodology. A further study underlining the aforementioned results in an in vivo mouse model was carried out by Yuan et al. by using a 10 wt% GelMA solution and combining it with human umbilical vein endothelial cells and human immortalized keratinocyte cell culture lines [36]. They were able to demonstrate a significant increase in cell migration by scratch assay, angiogenesis by tube formation assay and reepithelization rates in a wound model by IHC. Although not specifically investigated, we hypothesize that GelMA made the increase of the previously mentioned factors possible and likely enhances cell migration as well. More recently, we provided the first evidence for in vivo adipogenesis of mSVF and fibrin hydrogel in an advanced supermicrosurgical rat model [27]. DAPI/phalloidin staining revealed a visual increase in cell nuclei, actin filaments, cell elongation, and alignment, further indicating that GelMA may provide a conducive microenvironment that incorporates extracellular matrix components of mSVF.

Growth factors are believed to be a major mode of action of mSVF and adipose-derived progenitor cells. Secretion levels of bFGF, a marker for angiogenesis and cell growth in wound repair [37], were stable over 21 days indicating that cells within mSVF maintained their ability to secrete reparative growth factors.

Finally, the co-culture assay revealed the potential of the GelMA–mSVF to contribute to fibroblast proliferation, an important aspect of cutaneous wound healing. These results are consistent with a study by Rehman et al., demonstrating improved proliferation of fibroblasts, endothelial cells, and keratinocytes in an in vivo setting by a chicken embryo angiogenesis assay using GelMA as the loading platform [38]. It may be noted that the decrease in fibroblast proliferation over time is due to the starvation medium.

In terms of the mechanical properties of our hydrogel, we demonstrated a functionalization degree of 55% by NMR spectroscopy (Supplementary Figure S1). It is clear that the mixing of mSVF and GelMA significantly affects these properties including degradation rates. However, we refrained from investigating the mechanical characteristics of the composite materials given that all experiments have been conducted in the presence of mSVF and consequently due to the challenges of mechanical testing on samples with human tissue. One of our previous studies implemented 4 wt% resulting in hydrogel of 2 kPa [39]. We do not have any results in terms of our 7.5 wt% GelMA with the degree of functionalization mentioned above; yet, it should be in a similar order of magnitude. Moreover, we are confident that the swelling, biodegradability, and hemolysis characteristics are in line with the ones measured and described by Xia et al. The authors demonstrated a hemolysis rate of less than 5%, which indicates good blood compatibility. GelMA alone showed a swelling rate of around 18% and a remaining mass of roughly 4.5% over the course of 21 days [40]. However, combining GelMA with alginate did result in a significantly more stable construct with a remaining mass of circa 20%. The authors argued that this might be due to the increased viscosity and improved compressibility, both enhancing the stability of the hydrogel. As mentioned above, we did not specifically measure the mechanical properties of our constructs of mSVF and GelMA; nevertheless, these showed little to no macroscopic degradation after 21 days as illustrated in Figure 1, therefore maintaining integrity over the full time of the experiments. This could be due to the mix of ECM and cells of the mSVF enhancing the stability of the hydrogel by the already viscous tissue sample and the progressive cell proliferation over time. One could argue that the omission of an in-depth characterization of our GelMA-mSVF constructs in terms of mechanical properties is a limitation of our study; however, our primary goal was a feasibility study of the combination of mSVF and GelMA. Our construct demonstrated an enhancement in cell viability and proliferation was macroscopically stable over 21 days, which is a stepping stone for future mechanical characterization of mSVF-laden GelMA and in vivo investigations.

GelMA is cross-linked by exposure to UV light which may be clinically translatable to topical application in wounds. While our setup was designed for small GelMA–mSVF samples, larger UV lamps capable of covering larger surfaces appear to be necessary in clinical scenarios. Also, settings have to be adapted depending on the thickness and special needs of wounds, which could result in negative effects on mSVF cell properties. Currently, GelMA is not yet approved by the US Food and Drug Administration or the European Medicines Agency as a standalone therapeutic agent. As an encouraging fact, it is subject to investigation in various preclinical and clinical studies for a range of applications, including tissue engineering, drug delivery, and wound healing [10].

## 4. Materials and Methods

### 4.1. Tissue Collection

Protocols of our study were conducted in compliance with the Declaration of Helsinki. The collection of human samples was approved by the Ethical Committee of the Canton of Zurich, Switzerland (BASEC 2019-00389).

### 4.2. Sample Collection

Microfat from healthy subcutaneous depots was harvested during elective surgeries and directly transferred to the laboratory for further processing. Tissue from patients who were younger than 18 years of age and presented with a history of cardiovascular or autoimmune diseases, malignancies, morbidities or a pathological donor site area (scars, wound healing disorder, dermatologic disorders) was excluded.

### 4.3. Protocol for mSVF Isolation

Mechanical SVF isolation was performed according to a previously established protocol [9]. Lipoaspirates were first centrifuged at 1200× *g* for 3 min, followed by the removal of the upper oily fraction and lower watery fraction. The purified fat was emulsified using Luer-to-Luer connectors (Tulip Aesthetics^®^, San Diego, CA, USA) [41]. A second centrifugation with the removal of oily and watery fractions results in mSVF.

### 4.4. Synthesis of GelMA and LAP

Synthesis of GelMA and lithium phenyl-2,4,6-trimethylbenzoylphosphinate (LAP) was done according to established protocols [23].

For the synthesis of GelMA, drops of Methacrylic anhydride (MA, 12 g) are added to the gelatin solution, which is obtained by dissolving type-a gelatin in dH_2_O (150 mL) at 50 °C, and the reaction continues for 1.5 h at 50 °C. The solution is centrifuged at 3500 rcf for 5 min after it was transferred to a 50 mL tube. After decanting, the supernatant containing GelMA and the solution is diluted 1:2 by volume with warm dH_2_O (40 °C). This step is followed by a transfer to dialysis tubing (SnakeSkinTM dialysis tubing, 3.5 kDa MWCO; catalog number 88 244, Thermo Scientific, Waltham, MA, USA) as well as dialysis against dH_2_O for 7 days at 40 °C. Dialysis water was changed twice daily. The solution was then diluted 1:10 by volume with warm dH_2_O (40 °C), sterilized over a 0.2 µm filter, frozen at −80 °C overnight, and finally lyophilized for 5 days. Quantification of the degree of functionalization (ca. 70%) was carried out with 1H NMR(D_2_O) and with the calculated ratio of the lysine methylene signals (δ = 2.8–3.0 ppm) of GelMA to the phenylalanine signal (δ = 7.1–7.4 ppm) of unmodified gelatin.

For the synthesis of LAP, 2,4,6-trimethylbenzoyl chloride (3.2 g, 0.018 mL) and dimethyl phenylphosphonite (3 g, 0.018 mol) were mixed together slowly under argon and stirred at room temperature. After 18 h, a solution of lithium bromide (6.1 g, 0.072 mol) in 2-butanone (100 mL) was added to the mixture and heated to 50 °C for 10 min before cooling to room temperature. The resulting solution was filtered, washed with 2-butanone (100 mL) three times, and dried under vacuum. The quality of the product was confirmed to be of good quality via 1H NMR analysis (400 MHz, D_2_O).

### 4.5. Spectroscopic Analysis of GelMA

Spectroscopic analysis of the hydrogel was conducted by nuclear magnetic resonance (NMR) in the Macromolecular Engineering Laboratory at ETH Zurich and revealed a functionalization degree of 55%. The results are described in the Supplementary Materials section at the end of the manuscript and depicted in Supplementary Figure S1.

### 4.6. Protocol Establishment and Preliminary Experiments of GelMA-mSVF Mixture and Irradiation Time

Suitable settings for GelMA–mSVF mixes were established by several preliminary experiments. 

First, GelMA stock solutions (15 and 30 wt%) were mixed with mSVF in different ratios (GelMA:mSVF in 2:1, 1:1 and 1:2) in two rounds of experiments. Mixtures were then prewarmed at 37 °C and transferred (200 µL) to a slide that was covered with a silicon film (1 mm height) with a circular hole of 10 mm diameter. The GelMA-mSVF mix was then irradiated with an ultraviolet (UV) lamp (Thorlabs Inc., Newton, NJ, USA) to achieve crosslinking of GelMA by activating the photoinitiator (LAP) at a wavelength of 405 nm (S = 10 mW/cm^2^) for either 5 or 10 min. The resulting solidified GelMA-mSVF constructs were then transferred and incubated in a 12-well culture plate and a growth medium consisting of DMEM high glucose (VWR Chemicals, Radnor, PA, USA) + 10% Fetal Bovine Serum (Biowest SAS, Nuaillé, France) + 1% Penicillin-Streptomycin (Sigma Aldrich, St. Louis, MO, USA). The medium was changed every 3 days and after viability testing. Viability was measured on days 0, 1, 3, 7, 14 and 21 by AlamarBlue^®^ assay (AB; Life Technologies, Carlsbad, CA, USA) according to the manufacturer’s instructions. The assay ran for 24 h at 37 °C and was protected from any light sources. The supernatant was transferred to a 96-well plate in triplicates and viability was measured by fluorometry (λexc = 550 nm; λem = 600 nm) using a microplate reader (Cytation 5, BioTek Instruments, Winooski, VT, USA). Normalization of absorbance was achieved by using GelMA alone as a negative control and the medium as a blank. Irradiated mSVF alone served as a positive control.

**Figure 7:**
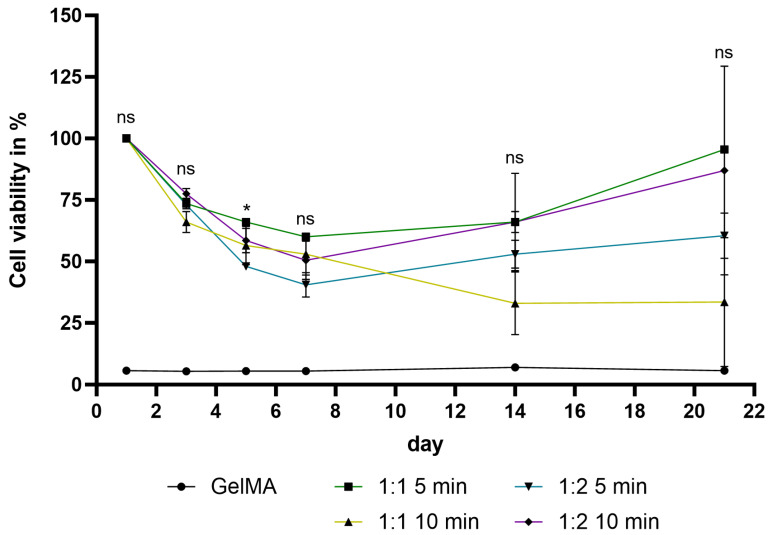
Viability assay of the first round of testing with different UV exposure times demonstrating a decrease of viability when exposure time was 10 min versus 5 min after 21 days. * signifies *p* ≤ 0.05. “ns” signifies non-significant.

The first round was mainly to establish the UV irradiation time (5 or 10 min) and its effects on the viability of the cells. During this round, only 15 wt% GelMA solution was used with a mixing ratio of 1:1 or 1:2 with mSVF. To summarize, 5 min of irradiation time proved to be the optimal setting in terms of level photocrosslinkage as well as cell viability over the course of 21 days. The difference was not statistically significant when comparing 5 and 10 min in the 1:1 mixing ratio group on day 21; however, cell viability was nonetheless better, as was the construct itself. The results are illustrated in Figure 7.

**Figure 8:**
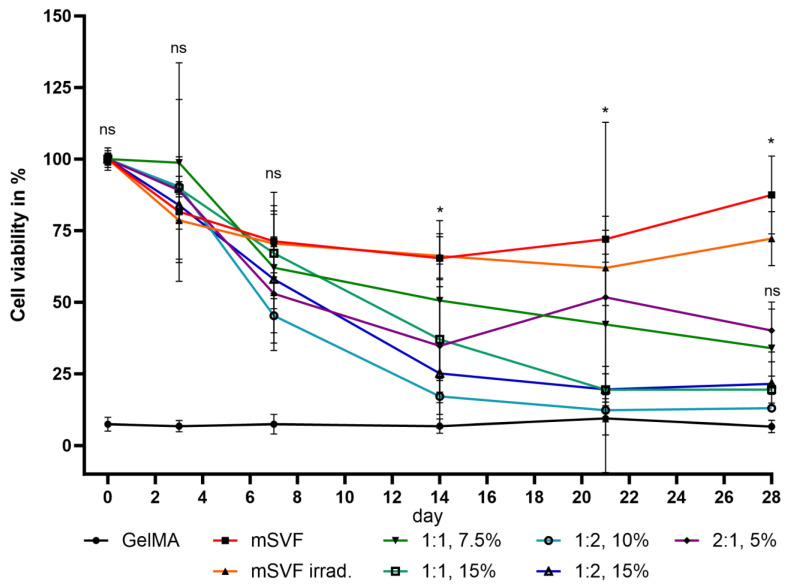
Viability assay of the second round of testing with different GelMA concentrations (using stock solutions of 15 and 30 wt%). A 1:1 mixture ratio of GelMA 15 wt% and mSVF, leading to an end concentration of 7.5 wt%, demonstrated the best combination of physical properties and cell viability. * signifies *p* ≤ 0.05. “ns” signifies non-significant.

The second round of testing included stock solutions of 15 and 30 wt% GelMA mixed in ratios of 1:1, 1:2 and 2:1 with mSVF. Exposure to UV light was 5 min for all samples, including negative (gel only) and positive controls, except for the non-irradiated positive control group. The positive control groups showed the highest viability overall which was significant when compared to all the other groups; however, more importantly, the optimal mixture resulting in the best degree of consistency, pliability and viability was achieved using a GelMA stock solution of 15 wt% and mixing it with mSVF in a 1:1 ratio with a final concentration of 7.5 wt%. The viability rates themselves, however, were not significantly higher. The viability rates are depicted in Figure 8.

### 4.7. GelMA-mSVF Mixture Protocol and UV Exposure Time

Cell viability, as determined using a metabolic activity assay (AlamarBlue^®^; Life Technologies, Carslbad, CA, USA) was quantified to identify desirable GelMA–mSVF ratios and UV irradiation times. Mixture of 100 µL 15 wt% GelMA with 100 µL mSVF (1:1) with an end concentration of 7.5 wt% GelMA and UV exposure time of 5 min (λ = 405 nm; I = 10 mW/cm^2^) exhibited promising cell viability and were used for all subsequent experiments.

**Figure 9:**
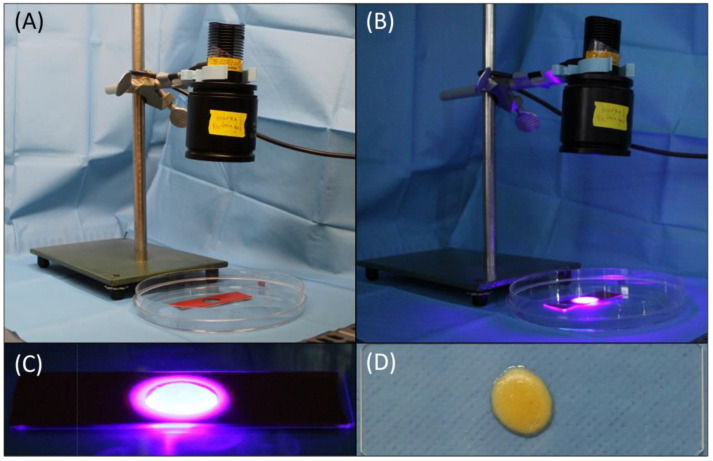
Setup in the laboratory including UV lamp, glass slide and the silicone mold (**A**,**B**) as well as the process of photocross-linking (**B**,**C**) and the resulting GelMA-mSVF construct (**D**), demonstrating physical evidence of stability.

After photopolymerization, solidified GelMA–mSVF samples were transferred to a 12-well culture plate and incubated in a growth medium at 37 °C for a total of 21 days. The setup in our laboratory (Figure 9A,B), including the process of cross-linking via UV light (Figure 9B,C), and the resulting construct (Figure 9D) are illustrated in Figure 9. The pipetting process of the mixture and initiation of cross-linking is found in Supplementary Video S1.

### 4.8. Viability Assay

Cell viability within GelMA–mSVF samples was measured on days 0, 1, 3, 7, 14, and 21 by AlamarBlue^®^ assay according to the manufacturer’s instructions and earlier protocol [42]. After treatment, the supernatant was transferred to a 96-well plate and absorbance was measured in triplicate using a microplate reader (Cytation 5, BioTek Instruments, Winooski, VT, USA). Normalization of absorbance was achieved by using GelMA alone as a negative control and the medium as a blank. Furthermore, irradiated mSVF without GelMA served as a positive control (PC).

### 4.9. ELISA

Culture medium was collected from the GelMA–SVF samples on days 1, 7, and 21, including from the PC group. The concentration of basic fibroblast growth factor (bFGF) in the medium was quantified by ELISA using a commercial human FGF-basic standard ABTS ELISA development kit and ABTS ELISA buffer kit (PeproTech, Cranbury, NJ, USA) according to the manufacturer’s instructions. Extinction was measured using a microplate reader mentioned above.

### 4.10. Histology, Immunohistochemistry (IHC) and Immunofluorescence (IF)

Tissue samples from GelMA–mSVF constructs were collected on days 7 and 21, stained by an automated IHC system, Autostainer Link48 (Agilent Dako, Santa, Clara, CA, USA), on previously formalin-fixed and paraffin-embedded tissue slices (3-μm thick). Slides were then processed using a PT Link device (Agilent Dako, Santa, Clara, CA, USA). The following antigens were targeted in addition to standard staining of hematoxylin eosin (HE) and trichrome staining: vascular endothelial growth factor A, cluster of differentiation 73 (Abcam, Cambridge, UK), and Perilipin-2 (Novus Biologicals, Centennial, CO, USA). 

Histopathology assessment was performed by two independent pathologists. Staining intensity was rated on a scale of 0 (negative), 1 (weak positive) or 2 (strong positive). Number of positive cells ranged from 0 to 100%.

Staining of cell nuclei and actin filaments was performed by IF using DAPI 300 µM (Cat.-Nr. A1001.0010, BioChemica, AppliChem, Darmstadt, Germany) and phalloidin (Cat.-Nr. ab176753, Abcam, Cambridge, UK) which was diluted in PBS in a ratio of 1:1000. Each GelMA–mSVF sample at days 0, 3, and 5 was stained with 100 µL of staining solution and incubated at room temperature for 90 min protected from light. Gels were then washed 3 times with 500 µL PBS for 5 min. Microscopy was performed using the THUNDER Live Cell imaging system (Leica Microsystems, Wetzlar, Germany).

### 4.11. Dermal Fibroblast Isolation and Co-Culture Assay

Human dermal fibroblasts were isolated from patient skin samples according to earlier protocols [43]. Co-culture assay was performed by seeding 70,000 primary human dermal fibroblasts per well (20,000 cells/cm^2^) with GelMA, GelMA–mSVF or alone as control. Gels were separated from fibroblasts through a well plate insert with a 40 µm mesh filter. Samples were incubated in a starvation medium (0.5% FBS) for 7 days. Viability was measured on days 0, 1, 3, and 7 again by AB assay.

### 4.12. Statistical Analysis

All values are presented as means with SEM. Normal distribution was tested by a Shapiro–Wilk test followed by an unpaired *t*-test with GraphPad Prism V8.0 (GraphPad Software, San Diego, CA, USA). *p* values  <  0.05 were accepted as statistically significant.

## 5. Conclusions

Our work indicates that GelMA is an intriguing and promising scaffold for mSVF. Mechanical SVF represents a mix of cells and ECM, and is an innovative cell source that can be easily harvested, processed, and applied for a multitude of ailments [44,45,46,47,48,49]. Our study is the first to investigate GelMA as a scaffold for mSVF in the broader field of tissue engineering. As a biocompatible scaffold, GelMA provided stable cell viability, proliferation, adipogenesis, stromal cell marker expression, and growth factor release. Importantly, GelMA–mSVF fosters the proliferation of dermal fibroblasts which may have implications for cutaneous wound repair.

## Figures and Tables

**Table 1:** Summary of patient demographics including age, gender and BMI.

Patient #	Age	Gender	BMI
1	40	Female	28.0
2	52	Female	30.9
3	18	Female	25.2
4	27	Female	31.2
5	48	Female	29.7
6	44	Female	21.6
7	34	Female	29.2
8	64	Female	34.6
9	65	Male	22.8
10	40	Female	25.6

## Data Availability

The data presented in this study are available within the manuscript and the Supplementary Materials Video S1 and Figure S1.

## Supplementary Materials

The following supporting information can be downloaded at: https://www.mdpi.com/article/10.3390/ijms241813944/s1.
