# Supplementary material for: Methacrylated Gelatin as a Scaffold for Mechanically Isolated Stromal Vascular Fraction for Cutaneous Wound Repair
Source: Int J Mol Sci. 2023 Sep 11;24(18):13944. doi: 10.3390/ijms241813944 (PMC10530931; doi:10.3390/ijms241813944)
Supplement: Supplementary file 1 [file ijms-24-13944-s001.zip › ijms-2568560-supplementary.pdf]

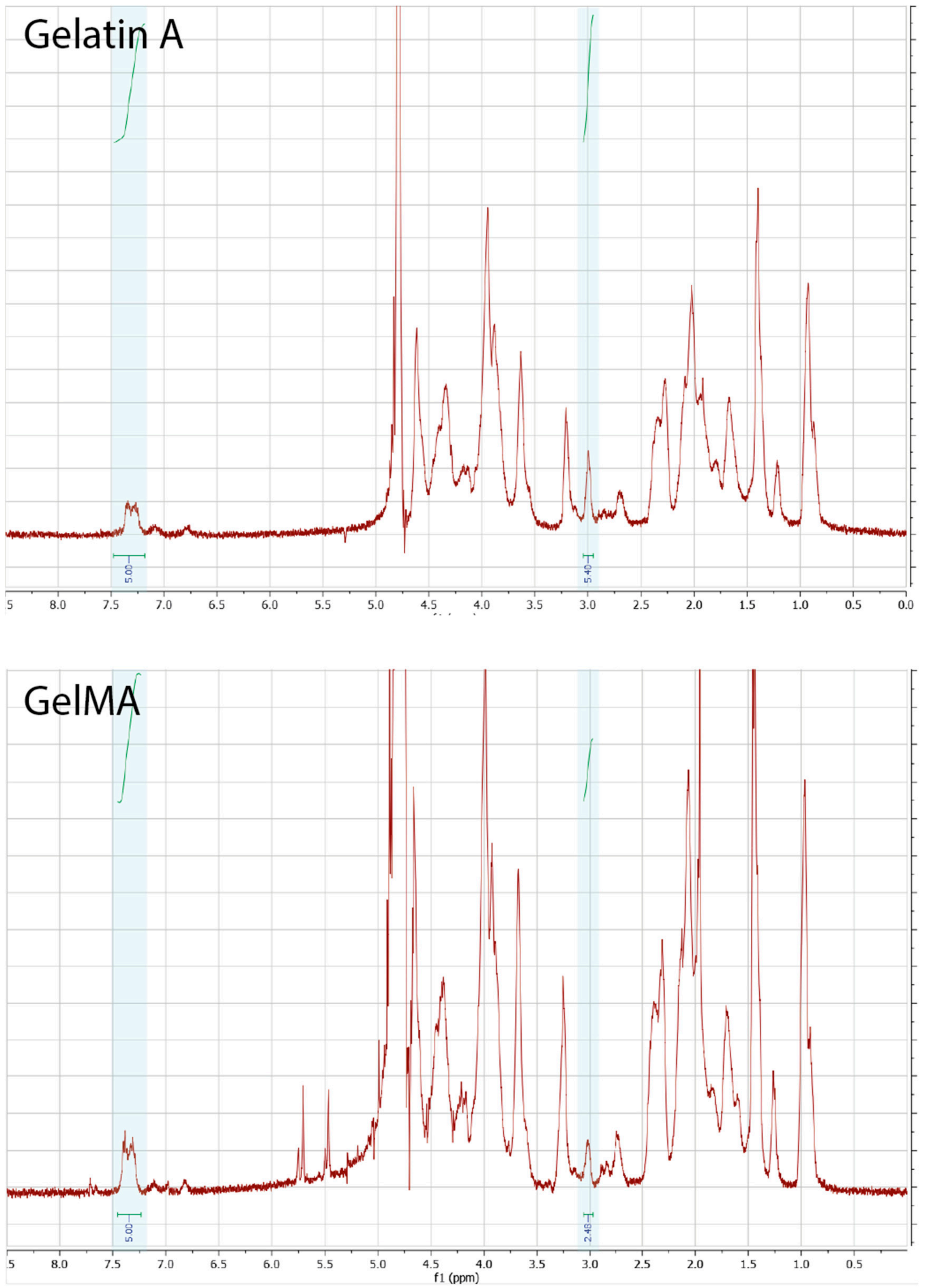

Figure S1: 1H NMR in D2O of gelatin (top) and GelMA (bottom). GelMA degree of functionalization (~55%) was quantified from the ratio of the integrated area of the lysine methylene signals (2.8–3.0 ppm) of GelMA and unmodified gelatin using the phenylalanine signal (7.1–7.4 ppm) as a reference.
